# Effect of a Single Intravesical Instillation of Mitomycin C on the Intravesical Recurrence Rate After Ureteroscopy for Upper Tract Urothelial Carcinoma: The SINCERE Prospective Multicenter Registry Study

**DOI:** 10.1016/j.euros.2025.03.017

**Published:** 2025-04-28

**Authors:** Orlane Figaroa, Ranan Dasgupta, Nora Hendriks, Guido Kamphuis, Jorg Oddens, Jeroen van Moorselaar, Adriaan Bins, Joyce Baard

**Affiliations:** a Department of Urology, Amsterdam UMC, University of Amsterdam, Amsterdam, The Netherlands; b Department of Medical Oncology, Amsterdam UMC, University of Amsterdam, Amsterdam, The Netherlands; c Cancer Center Amsterdam, Amsterdam UMC, Amsterdam, The Netherlands; d Department of Urology, Imperial College Healthcare NHS Trust, London, UK

**Keywords:** Intravesical recurrence, Mitomycin C, Upper tract urothelial cancer, Ureteroscopy

## Abstract

**Background:**

Upper tract urothelial carcinoma (UTUC) is a rare malignancy, accounting for 5–10% of all urothelial carcinomas (UCs). Radical nephroureterectomy (RNU) has been the standard treatment, but kidney-sparing surgery (KSS) via ureteroscopy (URS) is now recommended for low-risk cases. KSS is associated with a higher rate of intravesical recurrence (IVR). The SINCERE study is evaluating whether a single postoperative intravesical instillation of mitomycin C (SI-MMC) after URS can reduce the IVR rate.

**Study design:**

This is a prospective multicenter registry study enrolling patients with nonmetastatic UTUC undergoing URS followed by SI-MMC. Data will be compared to a historical control cohort without adjuvant MMC. Patients are aged ≥18 yr with no history of bladder cancer or contralateral UTUC. The study is following the principles of the Declaration of Helsinki and has received ethics approval.

**Primary and secondary outcomes:**

The primary outcome is total IVR and time to IVR. The secondary outcome is evaluation of predictive variables for IVR in patients with UTUC after endoscopic treatment.

**Discussion:**

Given the high recurrence rate after URS, the study aim is to provide evidence regarding MMC use to reduce IVR and address the current lack of robust data for this strategy. The study has potential to change clinical practice by demonstrating the efficacy of SI-MMC in preventing IVR after URS for UTUC.

## Take Home Message

SINCERE is a prospective multicenter study evaluating whether a single postoperative intravesical instillation of mitomycin C after diagnostic or therapeutic ureteroscopy for upper tract urothelial carcinoma can reduce the intravesical recurrence rate.

## 1 Background

Upper tract urothelial carcinoma (UTUC) is a rare condition [1]. Radical nephroureterectomy (RNU) has long been the golden standard treatment for UTUC. However, in cases of low-risk disease, treatment via ureteroscopy (URS) is now recommended by the European Association of Urology (EAU) and the American Urological Association as first-line treatment [2,3]. The obvious benefit of endoscopic treatment is the possibility of sparing renal units and thus preserve renal function. However several studies have revealed a higher rate of intravesical tumor recurrence (IVR) after endoscopic treatment via URS in comparison to RNU [[4], [5], [6], [7], [8], [9]]. The incidence of IVR following RNU is high at up to 46.2% according to literature reports [10]. A meta-analysis of available data showed a median IVR rate of 29%, with IVR occurring within a median time of 22.2 mo [11]. The IVR rate is even higher after endoscopic treatment, at up to 60% [12]. Furthermore, diagnostic URS in the workup for patients who undergo RNU is also associated with a higher IVR rate [5,[11], [12], [13], [14], [15]].

Mitomycin C (MMC) is the intravesical chemotherapeutic agent most commonly used in patients with UC of the bladder. MMC has an ablative effect (chemoresection) on residual tumor cells at the resection site and on small overlooked tumors after transurethral resection of bladder tumor [16]. Most importantly, chemotherapy destroys circulating tumor cells and prevents tumor cell implantation and is therefore used as an immediate single instillation (SI) after transurethral resection of bladder tumor or RNU for UTUC. Reports from a multicenter randomized study has shown the benefits of a single postoperative instillation of intravesical mitomycin C (SI-MMC) reducing the risk of IVR within the first year following RNU for UTUC with absolute risk reduction of 11% and a relative risk reduction of 40% [17]. Similar results were observed in a prospective randomized phase 2 trial after a SI of pirarubicin (THP), which reduced the recurrence rate to 16.9% at 1 yr and 2 yr in the THP group versus 31.8% at 1 yr and 42.2% at 2 yr in the control group [18].

Several predictors of IVR after RNU have been identified, leading to the hypothesis that IVR might be the result of a combination of intraluminal seeding and pan-urothelial disease [11]. During URS the chance of intraluminal tumor seeding towards the bladder is likely. It is possible that adjuvant SI-MMC after URS could lead to a reduction in IVR in this group of patients. However, as stated in the current EAU guidelines, there is currently no data to support the use of adjuvant intravesical instillation of chemotherapy after URS.

The aim of this prospective multicenter registry study is to evaluate whether a SI of MMC after URS for UTUC leads to a decrease in IVR.

## 2 Methods

### 2.1 Study design and eligibility criteria

**Fig. 1:**
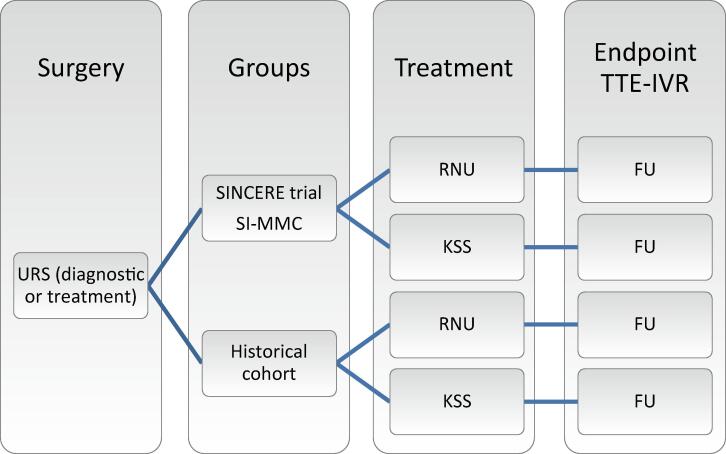
Overview of the SINCERE study design. FU = follow-up; IVR = intravesical recurrence; KSS = kidney-sparing surgery; RNU = radical nephroureterectomy; SI-MMC = single postoperative intravesical instillation of mitomycin C; TTE = time-to-event; URS = ureteroscopy.

SINCERE is a multicenter prospective registry in a clinical setting. Patients are eligible when aged ≥18 yr with clinically nonmetastatic UTUC and URS is planned for either diagnosis or treatment, followed by SI-MMC (or an equivalent agent) as an intravesical instillation, preferably within 24 h but no longer that 7 d after URS. Patient and tumor characteristics are collected. The primary objective is to analyze the effect of SI-MMC in the bladder on IVR in patients with UTUC and scheduled for therapeutic or diagnostic URS. A historical cohort will be used as a comparator. Fig. 1 gives an overview of the study design.

The standard dose of MMC is 40 mg in 50 ml of 0.9% NaCl according to the EAU guidelines on adjuvant treatment for bladder tumors and after RNU [2]. MMC is administered via a syringe connected to a transurethral catheter. The duration of the bladder instillation is 60 min up to a maximum of 2 h.

A history of bladder cancer, contralateral UTUC, and concurrent bladder cancer are the main exclusion criteria owing to the potential effect on IVR during follow-up. All the inclusion and exclusion criteria are listed in Table 1.

**Table 1:** Overview of the inclusion and exclusion criteria for the study

**Inclusion criteria**
(Suspicion for) nonmetastatic UTUC for which ureteroscopy is planned, for either diagnosis or treatment
Postoperative single instillation of mitomycin C (or any equivalent agent)
Age ≥18 yr
**Exclusion criteria**
History of histologically proven UC of the bladder, including carcinoma in situ
History of histologically proven UTUC in the contralateral kidney
Renal transplantation
Concurrent bladder tumor found preoperatively or perioperatively
Histology via intraoperative biopsies not feasible

UC = urothelial carcinoma; UTUC = upper urinary tract UC.

Follow-up data will be collected over 2 yr in accordance with the local protocol; as this is a registry study, there is no predefined follow-up schedule. During follow-up, cystoscopy and URS outcomes, including pathology results, will be collected.

### 2.2 Primary and secondary endpoints

The primary objective is to evaluate the IVR rate and the time to IVR after diagnostic or therapeutic URS followed by SI-MMC (or instillation of any equivalent agent) in patients with clinically nonmetastatic UTUC. The secondary objective is to evaluate variables that could potentially predict the development of IVR in patients with UTUC.

### 2.3 Sample size calculation and analysis plan

The estimated IVR rates after URS and RNU for UTUC are based on follow-up data collected between 2010 and 2020 in Amsterdam UMC.

• RNU treatment group: The recurrence-free survival (RFS) after 1 yr of follow-up is approximately 75%. Assuming that the treatment effect is similar to that observed from postoperative RNU data (reduction in the risk of bladder recurrence of 40%) [17], we require 70 participants in this cohort to achieve 80% power at a significance level of 0.05 to detect a hazard ratio of 0.565 when the control hazard rate is 0.288 using a two-sided log-rank test. The expected overall dropout rate is 2%.
• Kidney-sparing surgery (KSS) group: The RFS rate after 1 yr of follow-up is ∼65%. Assuming that the treatment effect is similar to that observed from postoperative RNU data (reduction in the risk of bladder recurrence of 40%) [17], we require 50 participants in this cohort to achieve 80% power at a significance level of 0.05 to detect a hazard ratio of 0.547 when the control hazard rate is 0.431 using a two-sided log-rank test. The expected overall dropout rate is 2%.

Parameters that follow a normal distribution will be reported as the mean ± standard deviation. Parameters that do not follow a normal distribution will be reported as the median and range. The IVR rate and time to IVR assessed at 2 yr after treatment via URS and adjuvant SI-MMC is the primary endpoint of the study. The IVR rate and time to IVR will be compared to recurrence data from a historical cohort.

The historical cases will be selected using the following criteria: age ≥18 yr; receipt of diagnostic or therapeutic URS; no lymph node or distant metastasis at the time of diagnosis, as assessed via computed tomography of the thorax and abdomen (cN0M0); a minimum of 2 yr of follow-up after surgery; no perioperative systemic chemotherapy; no history of histologically proven UC of the bladder, including carcinoma in situ (CIS); and no history of histologically proven UTUC in the contralateral kidney. The difference in recurrence rate between the intervention cohort and historical cohort will be assessed using Andersen-Gill and frailty models for survival analysis. The Andersen-Gill model is based on Cox regression for analyses of recurrent events, as standard survival and regression analysis models only consider the first event, which leads to a loss of information.

One of the secondary objectives of the study is to identify potential risk factors associated with clinical outcomes using multivariable Cox proportional-hazards analysis. This statistical approach will allow us to assess the independent impact of different factors on the IVR rate and time to IVR. The variables selected for inclusion in the model will be based on their clinical relevance and evidence from the literature. These include the timing of intravesical chemotherapy, histological grade of the tumor, location of the tumor, presence of concomitant CIS, tumor size, focality (unifocal or multifocal lesions), and procedural factors such as use of an access sheath or double-J stent placement during surgery.

### 2.4 Data handling and storage

All data will be handled with strict confidentiality and will be coded by each participating center to ensure privacy. A subject identification code list will be used to link the data to the corresponding participant, but the codes will not include patient initials or birth dates. A screening log will also be maintained for each participating center.

All data will be collected and securely stored in the Castor EDC database, an electronic data capture system designed to record information in prebuilt electronic case report forms. Data storage is safeguarded using certified servers, field-level encryption, and two-factor authentication, in compliance with Good Clinical Practice, Health Level 7 Fast Health Interoperability Resources standards, and other relevant regulatory guidelines (https://castoredc.com).

## 3 Discussion

Despite the widespread adoption of endoscopic management for UTUC, evidence from high-quality large-scale randomized controlled trials (RCTs) on the role of MMC is still lacking. This partly reflects the rarity of UTUC, and highlights the need for an accepted and agreed protocol for MMC use to demonstrate its benefit.

### 3.1 Necessity of the study

The role of SI-MMC as adjuvant therapy in UTUC to prevent IVR after endoscopic treatment remains unclear. The literature lacks high-quality evidence on the efficacy of MMC instillation following URS for UTUC.

The natural history of UTUC after URS treatment is characterized by a risk of recurrence, which can occur both in the upper tract and in the bladder. IVR may complicate management of these patients and leads to a need for invasive treatments such as transurethral resection of tumor and adjuvant bladder instillations. Given the high recurrence rate, interventions that could reduce the risk of IVR are highly desirable.

### 3.2 Rationale for a registry-based study design

Given the rarity of UTUC, recruitment of a sufficiently large cohort for an RCT is challenging and costly. Many centers may treat only a handful of UTUC patients who meet the inclusion criteria annually, making it difficult to gather large numbers of participants. In addition, because of the relatively low incidence of this disease, securing funding and grants for large-scale RCTs within a confined timeline is challenging. A registry-based study design offers a practical and efficient alternative, as it allows collection of real-world data across multiple institutions without a need for the intensive funding and the time and infrastructure required for an RCT.

A key strength of the registry approach is its ability to pool data from a variety of institutions, thereby increasing the sample size and improving the generalizability of the findings. By involving multiple centers internationally, the SINCERE study is leveraging the collective experience of a diverse group of clinicians and institutions. This not only enhances the external validity of the study but also ensures that the findings are applicable to a broad patient population. In rare diseases such as UTUC, for which the number of cases at any given institution is limited, this collaborative approach is essential to amass a large enough data set to draw meaningful conclusions.

The multicenter registry design also allows for a more inclusive patient population, encompassing a wide range of clinical scenarios and management approaches. By contrast, RCTs often have strict inclusion and exclusion criteria, which can limit their applicability to real-world practice. By capturing data from patients treated in routine clinical practice, the SINCERE study will provide valuable insights into the effectiveness of SI-MMC in a variety of clinical settings, which may better reflect the outcomes in day-to-day practice.

### 3.3 Challenges and limitations of registry-based studies

While registry-based studies offer several advantages, they also present unique challenges and limitations that must be acknowledged. One key limitation of this registry study is the lack of a prescheduled follow-up regimen. In an RCT, follow-up visits are typically standardized and scheduled at predetermined intervals, which ensures that data are collected in a consistent manner across all participants. By contrast, registry studies rely on routine clinical follow-up according to local protocols, which may vary between institutions and individual patients. This can introduce variability in the timing and frequency of follow-up, which may affect the accuracy and completeness of the data collected.

Another potential limitation of registry studies is the possibility of selection bias. Because registry studies do not involve randomization, there is a risk that patients who receive SI-MMC may differ from those who do not in ways that could influence the outcomes. For example, clinicians may be more likely to use SI-MMC in patients with higher-risk disease, which could skew the results. However, the prospective nature of the SINCERE study helps to mitigate this risk by ensuring that data are collected for all eligible patients, regardless of whether they receive SI-MMC or not. In addition, the large sample size and multicenter design should help in reducing the impact of any potential biases. Lastly, the proposed design includes centers that offer both RNU and KSS. It is expected that this will reduce bias, especially when cases are reviewed in multidisciplinary team meetings.

Despite these challenges, the registry-based design of the SINCERE study offers several important advantages that make it well suited to investigating treatment in a rare disease such as UTUC. By allowing collection of real-world data from a large, diverse patient population, the study will provide valuable insights into the effectiveness of SI-MMC in routine clinical practice. Moreover, the prospective nature of the study ensures that data are collected in a systematic and rigorous manner, which will enhance the reliability and validity of the findings.

### 3.4 Significance and novelty of the SINCERE study

The SINCERE study represents a significant and novel contribution to the field of UC research. To the best of our knowledge, this is the first multicenter, prospective, international study to specifically investigate the effect of SI-MMC on the IVR rate following URS for UTUC. This makes the study a landmark in the field, as it will provide the first high-quality evidence on this important clinical question.

The multicenter and international nature of the study is a key strength, as it allows for collection of data from a diverse group of patients and institutions. This will enhance the generalizability of the findings and ensure that the results are applicable to a wide range of clinical settings. The international collaboration involved in SINCERE highlights the importance of global efforts to improve the management of rare diseases such as UTUC. By bringing together researchers and clinicians from multiple countries, the study demonstrates the power of international collaboration in addressing challenging clinical questions that cannot be answered by single-center studies alone.

In addition to its novelty and significance, the SINCERE study also has the potential to influence clinical practice in a meaningful way. If the results demonstrate a significant reduction in the IVR rate with SI-MMC, this could lead to changes in the standard of care for patients with UTUC. There is currently no high-level evidence on the use of adjuvant therapies following URS for UTUC, and results from this study could provide the evidence needed to support routine use of MMC in this setting. This could ultimately improve outcomes for patients by reducing the risk of recurrence and the need for more invasive treatments.

## 4 Conclusions

The SINCERE study is a critically important investigation addressing an important gap in knowledge regarding the management of UTUC. The multicenter, prospective registry design allows for collection of real-world data from a large and diverse patient population, which is essential for studying a rare disease such as UTUC. While there are inherent challenges and limitations to registry-based studies, the prospective nature of the SINCERE study and the inclusion of multiple international centers can help in mitigating these concerns. As the first study of its kind, SINCERE has the potential to make a meaningful impact on clinical practice by providing the evidence needed to guide the use of SI-MMC following URS for UTUC. Ultimately, the study findings should help in improving care for patients with UTUC and reducing the burden of IVR in this challenging population.

  ***Author contributions***: Orlane Figaroa had full access to all the data in the study and takes responsibility for the integrity of the data and the accuracy of the data analysis.

  *Study concept and design*: Figaroa, Hendriks, Kamphuis, Oddens, van Moorselaar, Bins, Baard.

*Acquisition of data*: Figaroa, Hendriks, Baard.

*Analysis and interpretation of data*: Figaroa, Hendriks, Baard.

*Drafting of the manuscript*: Figaroa, Hendriks, Baard.

*Critical revision of the manuscript for important intellectual content*: Figaroa, Dasgupta, Hendriks, Kamphuis, Oddens, van Moorselaar, Bins, Baard.

*Statistical analysis*: Figaroa, Baard.

*Obtaining funding*: None.

*Administrative, technical, or material support*: None.

*Supervision*: Hendriks, Kamphuis, Oddens, van Moorselaar, Bins, Baard.

*Other*: None.

  ***Financial disclosures:*** Orlane Figaroa certifies that all conflicts of interest, including specific financial interests and relationships and affiliations relevant to the subject matter or materials discussed in the manuscript (eg, employment/affiliation, grants or funding, consultancies, honoraria, stock ownership or options, expert testimony, royalties, or patents filed, received, or pending), are the following: None.

  ***Funding/Support and role of the sponsor*:** None.

## 2.5 Ethics approval and consent to participate

The study will be conducted to the standards of Good Clinical Practice in full conformance with the Declaration of Helsinki (October 2013 version) and Dutch laws and regulations. Waiver of consent from the medical ethics commission of Amsterdam UMC (reference W22_369 #22.530) was obtained on December 23, 2022.
